# Marine Peptides and Their Anti-Infective Activities

**DOI:** 10.3390/md13010618

**Published:** 2015-01-16

**Authors:** Hee Kyoung Kang, Chang Ho Seo, Yoonkyung Park

**Affiliations:** 1 Department of Biomedical Science, Chosun University, Gwangju 501-759, Korea; E-Mail: hkkang129@gmail.com; 2 Department of Bioinformatics, Kongju National University, Kongju 314-701, Korea; E-Mail: chseo@kongju.ac.kr; 3 Research Center for Proteineous Materials, Chosun University, Gwangju 501-759, Korea

**Keywords:** marine organisms, anti-infective peptides, antimicrobial, antifungal, antiviral

## Abstract

Marine bioresources are a valuable source of bioactive compounds with industrial and nutraceutical potential. Numerous clinical trials evaluating novel chemotherapeutic agents derived from marine sources have revealed novel mechanisms of action. Recently, marine-derived bioactive peptides have attracted attention owing to their numerous beneficial effects. Moreover, several studies have reported that marine peptides exhibit various anti-infective activities, such as antimicrobial, antifungal, antimalarial, antiprotozoal, anti-tuberculosis, and antiviral activities. In the last several decades, studies of marine plants, animals, and microbes have revealed tremendous number of structurally diverse and bioactive secondary metabolites. However, the treatments available for many infectious diseases caused by bacteria, fungi, and viruses are limited. Thus, the identification of novel antimicrobial peptides should be continued, and all possible strategies should be explored. In this review, we will present the structures and anti-infective activity of peptides isolated from marine sources (sponges, algae, bacteria, fungi and fish) from 2006 to the present.

## 1. Introduction

Marine organisms are important sources of bioactive molecules that have been used to treat various diseases. Unusual marine environments are associated with chemical diversity, leading to a resource of novel active substances for the development of bioactive products [1,2,3,4].

Oceans, which cover more than 70% of the earth’s surface, represent an enormous resource for the discovery of potential therapeutic agents. Over the last several decades, numerous compounds have been found in marine organisms with interesting pharmaceutical activities [1,5,6]. Therefore, marine organisms are thought to be a potential source of essential and novel biologically active substances for the development of therapeutics. In particular, marine peptides have attracted a great deal of attention due to their potential effects in promoting health and reducing disease.

Despite tremendous progress in medicine, infectious diseases caused by bacteria, fungi, and viruses are still a major threat to public health. Their impact is particularly large in developing countries due to the lack of access to medicines and the emergence of widespread drug resistance. Increasing numbers of microbial pathogens that have acquiring antibiotic-resistance have resulted in the increase demand for novel and effective antimicrobial compounds. In particular, studies evaluating the anti-infective actions of marine peptides are attracting increased researchers’ interest, and marine peptides have been increasingly considered as anti-infective drugs. The diversity of the marine environment has provided a unique source of bioactive chemical compounds that could lead to potential new drugs candidates.

Marine peptides are specific protein fragments that in addition to acting as sources of nitrogen and amino acids have numerous potential physiological functions [7]. These peptides have been obtained from algae, fish, mollusk, crustacean, crab and marine bacteria and fungus. Bioactive marine peptides based on their structural properties, amino acid composition and sequences have been shown to display a variety of bioactivities such as anti-tumor, antiviral, anticoagulant, antioxidant, immunoinflammatory effects, and other medicinal properties [1,2,8,9].

In this review, we will present the structures and anti-infective activity of peptides isolated from the main marine organisms and microorganisms of interest (sponges, algae, bacteria, fungi and fish) from 2006 to the present.

## 2. Marine Organisms and Microorganisms

### 2.1. Sponges

Sponges are sessile marine filter feeders that have developed efficient defense mechanisms against foreign attackers such as viruses, bacteria, or eukaryotic organisms. Marine sponges are one of the richest sources of pharmacologically-active chemicals in marine environments. It has been suggested that many of the bioactive secondary metabolites isolated from sponges are produced by functional enzyme clusters, which originated from the sponges and their associated microorganisms [10,11]. More than 5300 different products have been identified in sponges and their associated microorganisms, and more than 200 new metabolites from sponges are reported each year [10]. As infectious microorganisms evolve and develop resistance to existing pharmaceuticals, the marine sponge provides novel leads against bacterial, viral, fungal and parasitic diseases. Many marine natural products have successfully advanced to the late stages of clinical trials. The chemical diversity of sponge products is remarkable. In addition to the unusual nucleosides, bioactive terpenes, sterols, peptides, alkaloids, fatty acids, peroxides, and amino acid derivatives (all of them frequently halogenated) have been described in sponges. The early appearance of sponges in evolution has given them a lot of time for the development of an advanced chemical defense system. The synthesis of secondary metabolites is regulated, depending on conditions that the sponge experiences. The huge variety of secondary metabolites in marine sponges and the complexity of the compounds and their biosynthetic pathways can be regarded as an indication of their importance for survival. As infectious microorganisms evolve and develop resistance to existing pharmaceuticals, marine sponges provide novel leads against bacterial, fungal, and viral diseases [10,11].

### 2.2. Algae

Algae are very simple chlorophyll-containing organisms composed of one cell or grouped together in colonies or as organisms with many cells, sometimes collaborating together as simple tissues [12]. Algae are heterogeneous group of plants with a long fossil history. Two major types of algae have been identified. Macroalgae (seaweeds) occupy the littoral zone, whereas microalgae are found in both benthic and littoral habitats, as well as throughout the ocean waters as phytoplankton [13]. Phytoplankton include numerous organisms, including diatoms (*Bacillariophyta*), dinoflagellates (*Dinophyta*), green and yellow-brown flagellates, and blue-green algae (cyanobacteria). As photosynthetic organisms, this group plays a key role in the productivity of oceans and constitutes the basis of the marine food chain [12].

Marine algae produce a cocktail of metabolites with interesting biological activities, including anti-infective, anti-inflammatory and anti-proliferative, and with potential commercial value [14,15,16,17]. Structures exhibited by these compounds go from acyclic entities with a linear chain to complex polycyclic molecules. Their medical and pharmaceutical applications have been investigated for several decades [18].

Marine macroalgae have been used as foods, especially in China and Japan, and as crude drugs for the treatment of many diseases, such as iodine deficiency (goiter, Basedow’s disease, and hyperthyroidism). Some seaweeds have also been used as a source of vitamins, treatment for various intestinal disorders, such as vermifuges, and as hypocholesterolemic and hypoglycemic agents [19].

Marine eukaryotic microalgae are known to produce numerous useful products, but have attracted little attention in the search for novel anti-infective compounds. Current reports mainly concern diatoms and cyanobacteria [20].

Diatoms are ubiquitous and constitute an important group within the phytoplankton community, as well as making an important contribution to the total marine primary production. These microalgae exhibit a characteristic golden-brown color, due to a high amount of xanthophyll fucoxanthin, which plays a major role in the light-harvesting complex of photosystems. In the water column, diatoms are exposed to rapidly varying light intensities. Diatoms produce an array of biologically active metabolites, many of which have been ascribed to a form of chemical defense, and which may have potential as candidate marine drugs [21].

Blue-green algae (cyanobacteria) show many structural features that are in common with bacteria. However, they are classified with algae because they contain chlorophyll a and related compounds. These algae are ancient photosynthetic prokaryotic organisms that produce biologically active secondary metabolites with diverse chemical structures such as nitrogenous compounds and cyclic polyethers [17]. Cyanobacteria may have evolved this extensive capacity to produce bioactive molecules because they are prokaryotes that have developed beyond a microscopic lifestyle, and hence require an arsenal of defensive substances to ward off predation by diverse macrograzers. Recently, several marine cyanobacterial natural products have been the focus of much attention, due to their intriguing structures and exciting biological activities [22].

### 2.3. Microorganism

Microorganisms have been the source of many valuable compounds in medicine, the pharmaceutical industry, and agriculture; however, most of these organisms are derived from terrestrial habitats. After intensive studies on terrestrial microorganisms, attention has now been focused on other ecosystems, such as the sea. Marine microorganisms, including bacteria and fungi, are rich sources of chemical products. Marine microorganisms are a promising new source of a large number of biologically active products [23,24,25,26]. Some of these marine species survive in a stressful habitat, under cold, lightless, and high-pressure conditions. These factors have resulted in the development of unique metabolisms, which result in the production of novel metabolites that differ from terrestrial organisms. Thus, marine microorganisms offer a wonderful resource for the discovery of new compounds with interesting biological activities, including antimicrobial, antifungal, anti-protozoan, anti-tuberculosis, and antiviral properties [27,28]. Thus far, only a small number of microorganisms have been investigated for their bioactive metabolites; however, a large number of active substances have been isolated, some of which feature unique structural skeletons.

### 2.4. Fish

Fishery products, including underutilized fish, jellyfish, and mollusks, also contain diverse and novel compounds. Some of these function as hypotensive agents, cardio-protective substances, muscle relaxants, and antimicrobial, antiviral, and anti-tumor agents [2,29,30].

Fish peptides exhibit broad-spectrum antimicrobial activity, killing both fish and human pathogens. These peptides have immunomodulatory effects, and the genes encoding these are highly responsive to microbes and innate immunostimulatory molecules. Recent research has demonstrated that some of the unique properties of fish peptides, including their ability to act even at very high salt concentrations, make them good potential targets for development as therapeutic antimicrobials [2,29,30].

In this review, we have surveyed peptides derived from marine sponges, algae, bacteria, fungi, and fish that have shown efficacy or activity against infectious and parasitic diseases, including bacterial, viral, fungal and protozoan infections.

## 3. Anti-Infective Marine Peptides

Table 1 presents new anti-infective peptides reported from 2006 to the present, along with their composition, source of origin, and reported bioactivity.

**Table 1:** List of anti-infective peptides from diverse marine sources.

Activity	Name of Peptide	Source of Original Peptides	Pharmacologic Activity	Inhibition Concentrations	References
Antibacterial	Aurelin (**1**)	Jellyfish: *Aurelia aurita*	*Escheichia coli* inhibition	7.7 μg/mL (MIC)	[31]
Antibacterial	Arenicin-1 (**2**)	Polychaete: *Arenicola marina*	*Pseudomomas aeruginosa* and *Staphylococcus aureus* inhibition	2 μg/mL (MIC)	[32,33]
Antibacterial	Tauramamide (**3**)	Bacterium: *Brevibacillus laterosporus*	*Enterococcus* sp. inhibition	0.1 μg/mL (MIC)	[34]
Antibacterial	Hepcidin (**4**)	Fish: *Oreochromis mossambicus*	*Listeria monocytogenes*,* S. aureus*, and *Enterococcus faecium* inhibition	50–100 μg/mL (MIC)	[35]
Antibacterial	Scygonadin (**5**)	Mud crab: *Scylla serrata*	*E. coli*,* P. aeruginosa*,* S. aureus*, *Streptococcus pyogenes* inhibition	50–100 μg/mL (MIC)	[36,37]
Antibacterial	Tunichromes (**6**)	Ascidian: *Ascidia nigra*	*Enterococcus* sp. inhibition	0.1 μg/mL (MIC)	[38,39]
Antibacterial	Bacillistatins 1 (**7**), 2 (**8**)	Bacterium: *Bacillus silvestris*	*Streptococcus. pneumonia* inhibition	0.5–2 μg/mL (GI_50_)	[40]
Antibacterial	Nacardiopsis thiopeptide TP-1161 (**9**)	Bacterium: *Nocardiopsis* sp	Vancomycin-resistant *Enterococcus faecium* inhibition	1 μg/mL (MIC)	[41]
Antibacterial	Centrocins 1 (**10**), 2 (**11**)	Sea urchin: *Strongylocentrotus droebachiensis*	*Corynebacterium glutamicum*, *S. aureus*,* Listonella anguillarum*, *E. coli* inhibition	1.3–5 μM (IC_50_)	[42]
Antibacterial	Halocyntin (**12**)	Ascidian: *Halocynthia papillosa*	*Micrococcus luteus*,* Bacillus megaterium*,* Aerococcus viridans*,* S. aureus*,* Enterococcus faecalis* inhibition	0.39–50 μM (MBC)	[43]
Antibacterial	Papillosin (**13**)	Ascidian: *Halocynthia papillosa*	*M. luteus*,* B. megaterium*,* Aerococcus viridans*,* S. aureus*,* Enterococcus faecalis* inhibition	0.05–6.25 μM (MBC)	[43]
Antibacterial	Hyastain (**14**)	Spider crab: *Hyas araneus*	*E. coli*, *Corynebacterium glutamicum*, *S. aureus* inhibition	0.4–12.5 μM (MIC)	[44]
Antibacterial	Indigoidine (**15**)	Bacterium: *Phaeobacter* sp.	*Vibrio fischeri* inhibition	ND	[45]
Antibacterial	Unnarmicins A (**16**), C (**17**)	Bacterium: *Photobacterium* sp.	*Pseudovibrio* sp. inhibition	7–18 μg/disk (disk)	[46]
Antibacterial	Ngercheumicins A–D (**18**–**21**)	Bacterium: *Photobacterium* sp.	Gram negative strain inhibition	ND.	[47]
Antibacterial	Solonamidine A (**22**), B (**23**)	Bacterium: *Photobacterium* sp	*S. aureus*, Methicillin-resistant *S. aureus* (MRSA)inhibition	ND.	[48]
Antibacterial	Cyclo-peptides (**24**)	Bacterium: *Pseudomonas* sp.	*S. aureus*, *M. luteus*, *B. subtilis*, *E. coli*,* V. anguillarum* inhibition	ND	[49]
Antibacterial	Ariakemicins A (**25**), B (**26**)	Bacterium: *Rapidithrix* sp.	*Bre*v*ibacterium* sp., *S. aureus*, *B. subtilis* inhibition	0.46–80 μg/mL (MIC)	[50]
Antibacterial	Damicornin (**27**)	Coral: *Pocillopora damicorins*	*M. luteus*, *B. megaterium*,* S. aureus*,* Brevibacterium stationis*,* Microbacterium maritypicum*,* Fusarium oxysporum* inhibition	1.25–20 μM (MIC)	[51]
Antibacterial	Clavanis (**28**)	Tunicate: *Styela clava*	*S. aureus*,* Klebsiella pneumonia*,* P. aeruginosa* inhibition	ND	[52]
Antibacterial	Cadiolides C–F (**29**–**32**)	Tunicate: *Pseudodistoma antinboja*	*S. aureus*,* S. epidermidis*,* Kocuria rhizophila and B. subtilis*, methicillin-sensitive* S. aureus* (MSSA), MRSAinhibition	0.13–12.5 μg/mL (MIC)	[53]
Antibacterial	Cytosporones B (**33**), E (**34**)	Fungus: *Leucostoma persoonii*	*S. aureus* USA100, MRSA, MSSA inhibition	72–78 μM (MIC)	[54]
Antibacterial	Anthracimycin (**35**)	Bacterium: *Streptomyces* sp.	*B. anthracis*,* Enterococcus facecalis*,* Streptococcus pneumonia*,* S. aureus*, MSSA, MRSA, vancomycin-resistant* S. aureus* inhibition	0.03125–0.25 μg/mL (MIC)	[55]
Antifungal	Halocidin (**36**)	Ascidian: *Halocynthia aurantium*	*Candida albicans* inhibition	1–4 μg/mL(MIC)	[56]
Antifungal	Callipeltine J (**37**), K (**38**)	Sponge: *Latrunculia* sp.	*C. albicans* inhibition	1 μM (MIC)	[57]
Antifungal	Pedein A (**39**)	Bacterium: *Chondromyces pediculatus*	*Rhizopus glutinis*, *Saccharomyces cerevisae*,* C. albicans* inhibition	0.6–1.6 μg/mL (MIC)	[58]
Antifungal	Theuellamide F (**40**), G (**41**)	Sponge: *Theonella* sp.	*C. albicans* inhibition	2–4.49 μM (IC_50_)	[59,60]
Antifungal	Theopapuamide B (**42**), C (**43**)	Sponge: *Siliquariaspongia mirabilis*	*C. albicans* inhibition	1–5 μg/disk (disk)	[61]
Antifungal	C(15)-surfactin (**44**)	Bacterium: *B. amyloliquefaciens*	*C. albicans* inhibition	0.004 μg/mL (MIC)	[62]
Antifungal	Anti-CAcyclic lipopeptide (**45**)	Bacterium: *B. amyloliquefaciens*	*C. tropicalis*, *Metschnikowia* *bicuspidata*, *Sacchromyces* *cerevisiae*, *Yarrowia lipolytica* inhibition	7.0 μg/mL (MIC)	[63]
Antifungal	Maribasins A (**46**), B (**47**)	Bacteriun: *B. marinus*	*Alternaria solani*,* Fusarium oxysporum*, *Verticillium alboatrum*,* F. graminearum*, *Sclerotium* sp., *Penicillium* sp., *Rhizoctonia solani*, *Colletotrichum* sp. inhibition	25–200 μg/mL (MIC)	[64]
Antifungal	Mojavensin A (**48**)	Bacterium: *B. mojavensis*	Phytopathogenic fungi inhibition	ND	[65]
Antifungal	Kahalalide F (**49**)	Mollusk: *Elysia rufescens*	*C. albicans*,* C. neoformans*,* Aspergillus fumigatus* inhibition	1.53–3.21 μM (IC_50_)	[66]
Antifungal	Miraenamide A (**50**), B (**51**)	Bacterium:* Paraliomyxa miuraensis*	*A. niger*,* Phytophthora capsici*,* Rhizopus oryzae*,* C. rugosa*, inhibition	0.4–25 μM (MIC)	[67,68]
Antifungal	Callyaerin A (**52**), E (**53**)	Sponge: *Callyspongia aerizusa*	*C. albicans* inhibition	5–10 μg/disk(disk)	[69]
Antimalarial	Dragomabin (**54**)	Bacterium: *Lyngbya majuscula*	*Plasmodium falciparum* W2 strain inhibition	6.0 μM (IC_50_)	[70]
Antimalarial	Venturamid A (**55**), B (**56**)	Bacterium: *Oscillatoria* sp.	*Plasmodium falciparum* W2 strain inhibition	5.6–8.2 μM (IC_50_)	[71]
Antimalarial	Aerucyamide A–D (**57**–**60**)	Bacteriun: *Microcystis aeruginosa*	*Plasmodium falciparum* K1 strain inhibition	0.7 μM (IC_50_)	[72,73]
Antimalarial	Gallinamide A (**61**)	Bacterium: *Schizothrix* sp.	*Plasmodium falciparum* W2 strain inhibition	8.4 μM (IC_50_)	[74]
Antimalarial	Lagunamide A (**62**), B (**63**)	Bacterium: *Lyngbya majuscula*	*Plasmodium falciparum* NF54 strain inhibition	0.19–0.91 μM (IC_50_)	[75]
Antimalarial	Albopunctatone (**64**)	Ascidian: *Didemnum albopunctatum*	*Plasmodium falciparum* Dd2, 3d7 strain inhibition	4.4–5.3 μM (IC_50_)	[76]
Antiprotozoal	Viridamide A (**65**), B (**66**)	Bacterium: *Oscillatoria nigro-viridis*	*Leishmania mexicana*,* Trypanosoma cruzi* inhibition	1.1–1.5 μM (IC_50_)	[77]
Antiprotozoal	Almiramides B (**67**), C (**68**)	Bacterium: *Lyngbya majuscula*	*Leishmania donovani* inhibition	1.9–2.4 μM (IC_50_)	[78]
Antiprotozoal	Valinomycin (**69**)	Bacterium: *Streptomyces* sp.	*Leishmania major* & *Trypanosoma brucei brucei* inhibition	0.0032–0.11 μM (IC_50_)	[79]
Antiprotozoal	Diketopiperazines (**70**–**81**)	Fungus: *A. fumigatus*,* Nectria inventa*	*Trypanosoma brucei*	0.002–40 μM (IC_50_)	[80]
Antituberculosis	Trichoderin A (**82**), A1 (**83**), B (**84**)	Fungus: *Trichoderma* sp.	*Mycobacterium tuberculosis* inhibition	0.02–2 μg/mL (MIC)	[81]
Antiviral	Mirabamides A (**85**), C (**86**), D (**87**), E–H (**88**–**91**)	Sponge: *Siliquariaspongia mirabilis*	Anti-HIV-1	0.041–3.9 μM (IC_50_)	[82]
Antiviral	Mollamides B (**92**)	Tunicate: *Didemmum molle*	Anti-HIV	48.7 μM (EC_50_)	[83]
Antiviral	Papuamide A (**93**)	Tunicate: *Didemmum molle*	Anti-HIV	71 nM (EC_50_)	[84]
Antiviral	Celebesides A (**94**), C (**95**)	Sponge: *Siliquariaspongia mirabilis*	Anti-HIV-1	1.9 μg/mL (IC_50_)	[61]
Antiviral	Theopapuamide A (**96**), D (**97**)	Sponge: *Theonella swinhoei*	Anti-HIV-1	0.5 μM (IC_50_)	[61,85]
Antiviral	Asperterrestide A (**98**)	Fungus: *Aspergillus terreus*	Anti-HIN1, Anti-H3N2	0.41–20.2 μM (IC_50_)	[86]
Antiviral	Homophymine A–E (**99**–**103**),A1–E1 (**104**–**108**)	Sponge: *Homophymia* sp.	Anti-HIV-1	75 nM (IC_50_)	[87,88]
Antiviral	Koshikmaide B (**109**), F–H (**110**–**112**)	Sponge: *Theonella* sp.	Anti-HIV-1	2.3 μM (IC_50_)	[89,90]

### 3.1. Antibacterial Activity

Several different organisms use antimicrobial peptides, typically 20–40 amino acids in length, for defense against infection. Most are capable of rapidly killing a wide range of microbes. Large antimicrobial proteins (>100 amino acids), are often lytic, nutrient-binding proteins or target specific microbial peptides by disrupting the structure or function of microbial cell membranes. A multitude of antimicrobial proteins have been found in the epithelial layers, phagocytes, and body fluids of multicellular animals, including humans. In addition to their role as endogenous antibiotics, antimicrobial peptides (AMP) contribute to inflammation, wound repair, and regulation of the adaptive immune system [91]. As part of ongoing global efforts to discover novel antimicrobials to treat infections caused by resistant pathogenic organisms, 35 studies have since 2006 reported novel antibacterial peptides isolated from marine sources such as bacteria, sponges, mud crabs, ascidin, spider crabs, jellyfish, and fish.

#### 3.1.1. Aurelin

**Figure 1:**
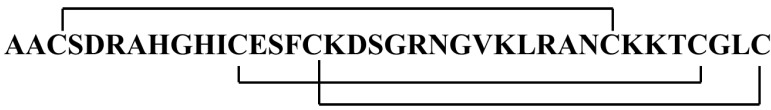
Amino acid sequence of aurelin (**1**). Aurelin was isolated from the mesoglea of a scyphoid jellyfish, *Aurelia aurita* [31]. Aurelin has six cysteine residues, forming three disulfide bonds.

Ovchinnikova* et al.* reported in the purification of a 40-residue AMP from the mesoglea of a scyphoid jellyfish, *Aurelia aurita*, Linnaeus, 1758 [31] (Figure 1). The peptide was named aurelin (**1**), and exhibited activity against gram-positive and gram-negative bacteria. Minimum inhibitory concentrations (MIC) were defined under low salt conditions (10 mM sodium phosphate buffer, pH 7.4). Aurelin inhibited the growth of microorganisms (MICs: 22.64 μg/mL against *Listeria monocytogenes* and 7.66 μg/mL against *Escherichia coli*). Interestingly, aurelin showed no sequence homology to any previously identified AMP, but displayed partial similarity to both defensins and K-channel-blocking toxins of sea anemones [31].

#### 3.1.2. Arenicin-1

**Figure 2:**
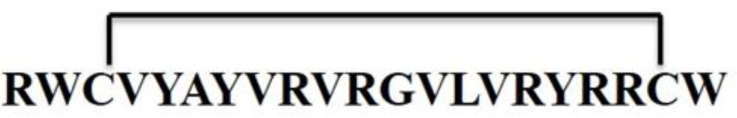
Amino acid sequence of arenicin-1 (**2**). Arenicin was isolated from the marine polychaete *Arenicola marina* [32]. Arenicin-1 contained one disulfide bond and formed a typical β-hairpin structure.

Lee* et al.* reported that the 21-residue peptide arenicin-1 (**2**), isolated from the marine polychaete *Arenicola marina*, exhibited significant antibacterial activity against *Pseudomonas aeruginosa* and *Staphylococcus aureus* (MIC = 2 μg/mL) [32]. Arenicin-1 contained one disulfide bond (Cys_3_-Cys_20_), and formed a typical β-hairpin structure [33] (Figure 2). Arenicin-1 can disrupt the cell membrane through the formation of highly oligomerized states, which cause pore formation [92]. These results emphasize the inherent difficulty in predicting the antimicrobial potential and the mode of action of a rather simple scaffold. Furthermore, arenicin-1 induced the release of calcein from PE/PG liposomes, thus suggesting that the bacterial cell membrane is the main molecular target of the peptide [33].

#### 3.1.3. Tauramamide

Tauramamide (**3**) is produced by cultures of the marine bacterial isolate *Brevibacillus laterosporus* PNG276, obtained from Papua New Guinea [34]. Tauramamide is a new lipopeptide antibiotic that contains two d-amino acids and is acylated at the *N*-terminus, two hallmarks of a non-ribosomal peptide synthase biosynthetic origin. Tauramamide showed potent (MIC = 0.1 μg/mL) and relatively selective inhibition of the gram-positive human pathogen *Enterococcus* sp. Moreover, tauramamide showed weak activity against Methicillin resistant *S. aureus* (MRSA; MIC = 200 μg/mL), although it was not appreciably active against *Candida albicans* (MIC = 50 μg/mL).

#### 3.1.4. Hepcidins

**Figure 3:**
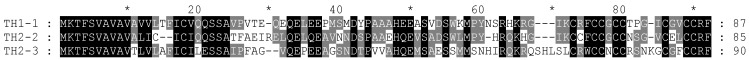
Sequence alignment of three tilapia hepcidins (TH1-5, TH2-2, and TH2-3). Three hepcidins (**4**) were isolated from tilapia (*Oreochromis mossambicus*) [35]. Identical or similar amino acid residues are in same colors. Gaps are inserted to obtain maximum homology.

Hepcidins (**4**) are cysteine-rich peptides isolated from tilapia (*Oreochromis mossambicus*), with MICs (50–100 μg/mL) against *L. monocytogenes*, *S. aureus*, and *Enterococcus faecium* [35]. Hepcidins are antimicrobial peptides that play important roles in resisting pathogenic infection. Through hybridization of a phage library, the cDNA sequences of three hepcidin-like antimicrobial peptides (named TH1-5, TH2-2, and TH2-3) were identified in tilapia (Figure 3). The predicted molecular weights of TH1-5, TH2-2, and TH2-3 are 9.5, 9.4, and 9.8 kDa, respectively. Fish hepcidins are active against a wide variety of bacteria, both gram-positive and -negative in the low μM range, including potent activity against a large number of fish pathogens. In contrast, their quantified activity against fungi appears to be rather low [35].

#### 3.1.5. Scygonadin

**Figure 4:**
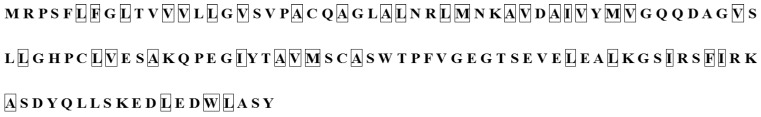
Amino acid sequence of scygonadin (**5**). Scygonadin was isolated from the seminal plasma of the mud crab, *Scylla serrate* [36,37]. Scygonadin contained α-helices and had 39 residues on the same hydrophobic surface. Scygonadin may interact with cell membranes.

Scygonadin (**5**) is an anionic antimicrobial peptide recently identified from the seminal plasma of *Scylla serrata*. [36,37]. This molecule (Figure 4), which showed no homology to any other protein in databanks, was able to inhibit *Micrococcus luteus* growth [93]. Scygonadin showed activity against *E. coli* (MIC = 25–50 μg/mL), *P. aeruginosa* (MIC = 12.5–25 μg/mL), *S. aureus* (MIC = 50–100 μg/mL) and *Streptococcus pyogenes* (MIC = 25–50 μg/mL). To gain more detailed information about its antimicrobial activity, the mature scygonadin peptide was expressed in *E. coli* to obtain a large quantity of the biologically active product. An approximately 43-kDa fusion protein, CKS-scygonadin, was obtained, and was highly stable and active [37].

#### 3.1.6. Tunichromes

Tunichromes (**6**) are small peptides containing one or more dehydrodopa-derived units that have been identified in several species of tunicates. Incubation of tunichromes isolated from *Ascidia nigra* hemocytes under oxidative conditions possess inherent crosslinking properties* in vitro* [38]. Three tunichromes have also been isolated from the blood cells of *Ascidia nigra* (An-1, An-2, An-3) [39]. Tunichromes show potent (MICs = 0.1 μg/mL) and relatively selective activity against the gram-positive human pathogen *Enterococcus* sp. [38].

#### 3.1.7. Bacillistatins 1 and 2

Two new cyclodepsipeptides, designated bacillistatins 1 (**7**) and 2 (**8**), have been isolated from cultures of a sample of *Bacillus silvestris* that was obtained from a Pacific Ocean (southern Chile) crab. Each 12-unit cyclodepsipeptide strongly inhibited growth in a human cancer cell line panel, with a 50% growth inhibition values (GI_50_s) of 10^−4^–10^−5^ μg/mL. Further, each compound was active against antibiotic-resistant *Streptococcus pneumoniae* [40].

#### 3.1.8. Thiopeptide TP-1161

The thiopeptide TP-1161 (**9**) was identified in a Norwegian marine sediment-derived gram-positive bacterium, *Nocardiopsis* sp. [41]. The* in vitro* antibacterial activity of TP-1161 was assessed against a panel of gram-negative and gram-positive bacteria, mostly representing clinical isolates. All of the gram-negative strains tested (six *E. coli*, two *Klebsiella pneumoniae*, one *Salmonella enterica* serovar Choleraesuis, and four *P. aeruginosa*) were not susceptible to TP-1161. However, the MICs against gram-positive strains ranged from 0.25 to 4 μg/mL, which was comparable to or lower than those of the reference antibiotic vancomycin. TP-1161 also inhibited the growth of vancomycin-resistant bacterial strains, including *Enterococcus faecalis* 560 and *Enterococcus faecium* 569, with an MIC of 1 μg/mL.

#### 3.1.9. Centrocins

**Figure 5:**
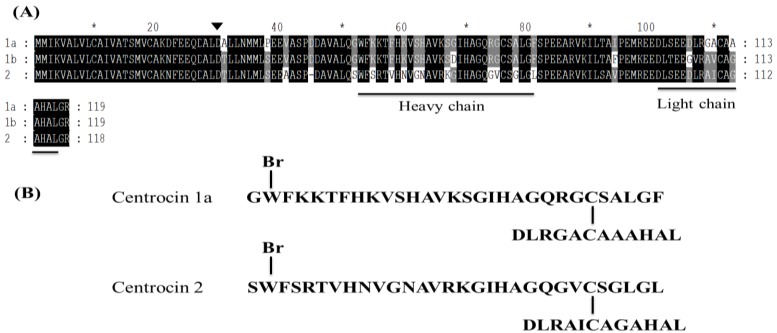
The alignment of centrocin 1 (**10**) and 2 (**11**) from *Strongylocentrotus droebachiensis* (**A**) and the proposed structure of centrocins 1a and 2 (**B**) [42]. The predicted cleavage site between the signal peptides and the prosequences are shown by a solid triangle (▼). Identical residues are shaded in black, whereas similar residues are shaded in gray. The boxes indicate the heavy chain and the light chain regions. In the proposed structure of centrocin, the heavy chain and the light chain are connected by disulfide bridges. The brominated tryptophan in position 2 of the active centrocin is labeled with a Br on the top.

Two novel AMPs, named centrocin 1 (**10**, 4.5 kDa) and 2 (**11**, 4.4 kDa), were purified from coelomocyte extracts of the green sea urchin, *Strongylocentrotus droebachiensis* (Figure 5). The native peptides are cationic and show potent activity against gram-positive (*Corynebacterium glutamicum*, *S. aureus*; IC_50_ = 1.3–5 μM) and gram-negative (*Listonella anguillarum*, *E. coli*; IC_50_ = 1.3–2.5 μM) bacteria. The centrocins have an intramolecular heterodimeric structure containing a heavy chain (30 amino acids) and a light chain (12 amino acids) [42].

#### 3.1.10. Halocyntin and Papillosin

**Figure 6:**
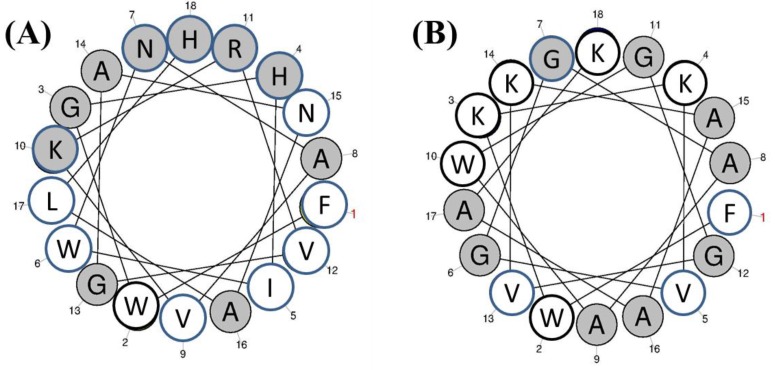
Helical wheel diagrams of halocyntin (**A**, **12**) and residues 2-19 of papillosin (**B**, **13**) are shown, with the polar residues shaded gray. The clearly evident clustering of polar and apolar residues imparts amphipathicity [43].

Halocyntin (**12**) and papillosin (**13**) are two new peptides (MIC = 0.75–25 μM) isolated from hemocytes of the Mediterranean ascidian *Halocynthia papillosa* [43]. Papillosin (GFWKKVGSAAWG GVKAAAKGAAVGGLNALAKHIQ, 34 amino acids residues) is longer than halocyntin (FWGHIWNAVKRVGANALHGAVTGALS, 26 amino acids residues), and also has a higher net charge (Figure 6).

Halocyntin (**12**) had marked activity against gram-positive strains, particularly *Micrococcus luteus*, *Bacillus megaterium*, and *Aerococcus viridans* (0.39 μM < minimum bactericidal concentration [MBC] < 1.56 μM). It also showed activity against *S. aureus* and *Entrococcus faecalis* (1.56 μM < MBC < 3.13 μM). Halocyntin activity against gram-negative bacteria was significantly lower, with MBCs between 6.25 and 50 μM, with the exception of *Klebsiella pneumoniae* that had MBC betwwen 1.56 μM and 3.13 μM [43].

Papillosin (**13**) showed important antibacterial activity against both gram-positive and gram-negative bacteria. Growth inhibition of gram-positive bacteria was particularly strong for *M. luteus*, *B. megaterium*, and *Aerococcus viridans* (0.05 μM < MBC < 0.39 μM), whereas *S. aureus* growth was less affected (1.56 μM < MBC < 3.13 μM). In contrast to halocyntin, papillosin also has strong activity against gram-negative bacteria (0.39 μM < MBC < 1.56 μM), with the exception of *P. aeruginosa* (3.13 μM < MBC < 6.25 μM) [43].

Papillosin possesses potent activity against gram-positive and gram-negative bacteria. Halocyntin is active against all the gram-positive bacteria tested, but its activity against gram-negative bacteria is effective but much less potent. In addition, for all strains tested, papillosin present a higher antibacterial activity than halocyntin.

#### 3.1.11. Hyastatin

**Figure 7:**
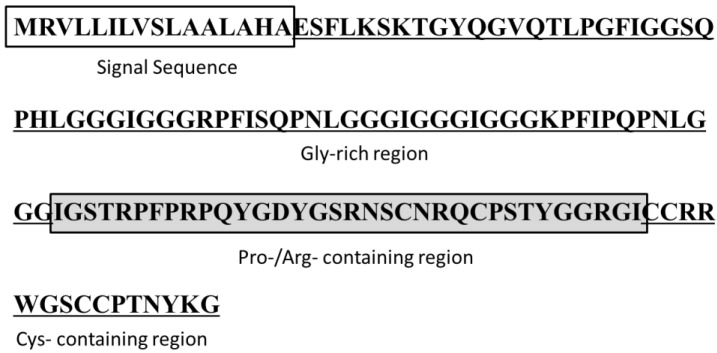
Amino acid sequences and overall peptide structure of hyastatin (**14**), isolated from the hemocytes of *Hyas araneus* [44]. The different regions are distinguished with boxes and given a designation below. The sequence of hyastatin has been submitted to the NCBI GenBank database with the accession number FJ764995.

Hyastatin (**14**) is a glycine-rich multi-domain peptide from hemocytes of the Norwegian spider crab *Hyas araneus* [44]. It consists of three distinctly different domains: an *N*-terminal region enriched in Gly residues, a short Pro/Arg-rich region, and a *C*-terminal region containing six Cys residues with a Cys pattern resembling the one found in penaeidins (Figure 7). The hyastatin transcript is constitutively expressed, and is primarily found in hemocytes. Hyastatin was purified in its native form *Hyas araneus* hemocytes and its antimicrobial potential was estimated against selected bacteria and fungi. The MIC value was defined as 50% growth inhibition compared to the growth control, and the highest concentration tested was 50 μM. Hyastatin had moderate activity against *E. coli* (MIC = 12.5 μM), but showed no activity against *P. aeruginosa*. The activity against gram-positive bacteria was either very strong or absent, with MIC values of 0.4 μM and >50 μM against *Corynebacterium glutamicum* and *S. aureus*, respectively [44].

#### 3.1.12. Indigoidine

A blue pigment is associated with *Phaeobacter* sp. Y4I-mediated inhibition of *Vibrio fischeri* [45]. This pigment, referred to as indigoidine (**15**), is produced by the condensation of two glutamine residues via a nonribosomal peptide synthase-based biosynthetic pathway. The indigoidine biosynthetic genes are *igiB*, which codes for a 6-phosphogluconate dehydrogenase, *igiC*, for a glutamate racemase, and *igiD* for indigoidine synthase [94].

#### 3.1.13. Unnarmicins

Unnarmicins are depsipeptides isolated from the fermentation broth of a marine bacterium *Photobacterium* sp. MBIC06485. Unnarmicins A (**16**) and C (**17**) contain two Leu, and two Phe. The difference between the two peptides was due to replacement of the 3-hydroxyoctanoyl moiety in A (**16**) with the 3-hydroxyhexanoyl group in C (**17**) [46]. These compounds exert antibacterial activity against only one species in the *Pseudovibrio* genus, a common α-Proteobacteria genera in the marine environment [46]. The antibacterial activity of unnarmicins against two gram-positive bacteria (*Firmicutes*) and seven halophilic gram-negative bacteria belonging to Bacteroidetes, Alphaproteobacteria, and Gammaproteobacteria was evaluated by a plate diffusion assay [46].

#### 3.1.14. Ngercheumicins

Depsipeptides from Photobacterium strains that are active against the non-pathogenic *Pseudovibrio denitrificans* were patented in 2007, and called ngercheumicins [47]. To date, five depsipeptides, ngercheumicins A–E, have been purified and characterized. Ngercheumicins A (**18**) and B (**19**) have a depsipeptide macrocycle containing one Phe and two Leu residues with different fatty acid tails. Ngercheumicins C (**20**) and D (**21**) have a macrocycle composed of three Leu, two Thr, and one Ser, with no fatty acid tails [47]. Although these compounds only target bacteria for which no pathologies have been described to date, ngercheumicins can be added to culture media to enhance the proliferation of slow-growing marine bacteria.

#### 3.1.15. Solonamides

Marine *Photobacterium* produce cyclodepsipeptides, called solonamides. Various depsipeptides have been identified in this genus, suggesting that this type of compound may be a common feature in *Photobacterium* [48]. No antibacterial activity has been observed for solonamides A (**22**) and B (**23**), although the activity has only been assessed against *Vibrio anguillarum* and *S. aureus*. Nevertheless, solonamide B (**23**) has been reported to reduce the expression of MRSA *hla* and *rnaIII*, two genes involved in strain virulence, controlled by an *agr*-dependent quorum sensing system. The structural similarity of the solonamides and the auto-inducing peptides involved in *S. aureus* quorum sensing suggests that solonamides may be competitive inhibitors of the *agr* system [48]. The reduced activity of solonamide A compared with solonamide B indicates that the overall hydrophobicity of the depsipeptides, due to the fatty acid chain, may have an important impact on inhibiting the expression of virulence genes in *S. aureus*.

#### 3.1.16. Cyclo-Peptides

A *Pseudoalteromonas* sp. associated with the sponge *Halisarca ectofibrosa* has been isolated for its ability to inhibit *S. aureus*, *M. luteus*, *B. subtilis*, *E. coli* and *V. anguillarum* [49]. The resulting fermentation broth contains four cyclo-peptides (**24**): cyclo-(Phe-Pro-Leu-Pro), cyclo-(Leu-Pro)_2_, cyclo-(Phe-Leu)_2_, and cyclo-(Leu-Ile)_2_ [49].

Although the fermentation broth extracted with methanol inhibits several target bacteria, no inhibitory activity is observed when cyclo-peptides are used alone. However, employing a solid phase synthesis method, the cyclo-(Phe-Pro-Leu-Pro) has been shown to exert a significant inhibitory activity against gram-negative bacteria, including *P. aeruginosa* and *Klebsiella oxytoca* [95]. The biosynthetic pathways involved in the synthesis of cyclo-peptides have not been investigated. The structural analogy with other tetrapeptides, however, suggests that NRPS modules are at the origin of this peptide, as in *Streptomyces* [96].

#### 3.1.17. Ariakemicins

Ariakemicins A (**25**) and B (**26**) are unusual, linear, hybrid, polyketide-nonribosomal peptide antibiotics from a marine gliding bacterium of the genus *Rapidithrix* [50]. The ariakemicins were composed of threonine, two Ω-amino-(Ω-3)-methyl carboxylic acids with diene or triene units, and δ-isovanilloylbutyric acid. The ariakemicins were tested against a panel of microbial strains consisting of three gram-positive bacteria (*Brevibacterium* sp., *S. aureus*, and *B. subtilis*: 83 μg/mL), four gram-negative bacteria (*Cytophaga marinoflava*, *Pseudovibrio* sp., *E. coli*, and *P. aeruginosa*: 83 μg/mL), and a yeast (*C. albicans*). The antibiotic mixture selectively inhibited the growth of gram-positive bacteria, among which *S. aureus* (0.46 μg/mL) was the most affected. The antibiotics had slight cytotoxicity against A549 human lung cancer cells and BHK baby hamster kidney cells with IC_50_ values of 25 and 15 μg/mL, respectively [50].

#### 3.1.18. Damicornin

**Figure 8:**
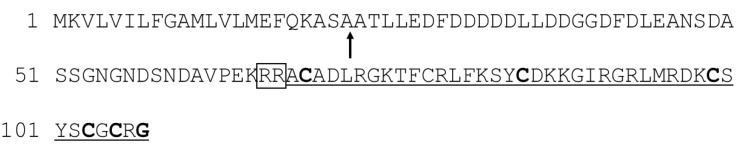
Deduced amino acid sequences of preprodamicornin. The arrow identifies the cleavage site of the signal peptide. The dibasic cleavage site between the acidic *N*-terminal proregion and the cationic *C*-terminal region is outlined in black. The damicornin active peptide is underlined in black. The cysteine residues and glycine amidation signal are shown in bold [51].

Vidal-Dupiol* et al.* reported that the 4492.35 Da peptide amicornin (**27**) from a scleractinian coral of *Pocillopora damicornis* [51]. Its precursor has a segmented organization comprising a signal peptide, an acidic proregion, and the *C*-terminal AMP. The 40-residue AMP is cationic, *C*-terminally amidated, and characterized by the presence of six cysteine molecules joined by three intramolecular disulfide bridges. Its cysteine array is common to another AMP and toxins from cnidarians [51] (Figure 8). Damicornin had antimicrobial activity against Gram-positive bacteria (*M. luteus*, *B. megaterium*, *S. aureus*, *Brevibacterium stationis* and *Microbacterium maritypicum*; MIC = 1.25–20 μM) and the filamentous fungus *Fusarium oxysporum* (MIC = 20 μM) but had little activity against Gram-negative bacteria except *E. coli* (MIC = 20 μM). In addition, the gene for expression of damicornin is repressed concomitantly with the invasion of host ectodermal cells by the coral pathogen *Vibrio coralliilyticus* [51].

#### 3.1.19. Clavanins

Clavanins (**28**) are antimicrobial peptides isolated from the marine tunicate *Styela clava*, showing 23 amino acid residues in length, cationic properties, and also high bactericidal activity [52]. In spite of clear benefits from the use of peptides, currently 95% of peptide properties have limited pharmaceutical applicability, such as low solubility and short half-life in the circulatory system. Saude* et al.* was reported that nanostructured clavanin A form shows improved antimicrobial activity and has the potential to be used to treat polymicrobial infections [52]. *In vitro* bioassays showed that the nanostructured clavanin was partially able to control development of *S. aureus*, *Klebsiella pneumoniae* and *P. aeruginosa*. *In vivo* sepsis bioassays were performed using C57BL6 mice strain inoculated with a polymicrobial suspension. Assays led to 100% survival rate under sub-lethal sepsis assays and 40% under lethal sepsis assays in the presence of nanoformulated clavanin A until the seventh day of the experiment [52].

#### 3.1.20. Cadiolides

**Figure 9:**
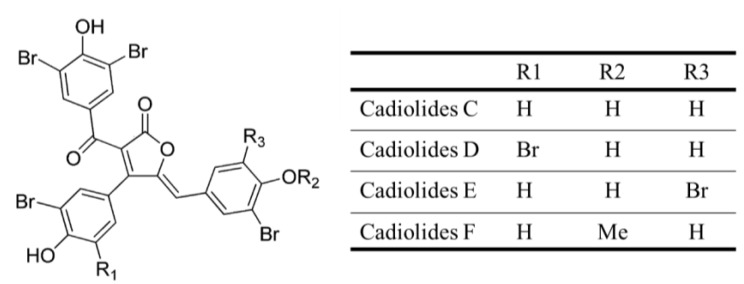
Structure of Cadiolides (**29**–**32**). Caldiolides were isolated from the tunicate *Pseudodistoma antinboja* by activity-guided fractionations [53].

Cadiolides C–F (**29**–**32**), butenolide metabolites (Figure 9), were isolated from the tunicate *Pseudodistoma antinboja* by activity-guided fractionations [53]. These compounds were evaluated for their antibacterial activity, and most of them exhibited moderate to significant activity that selectively targeted Gram-positive strains (*S. aureus*, *S. epidermidis*, *Kocuria rhizophila* and *B. subtilis*; MIC = 0.2–12.5 μg/mL). However, they did not show inhibitory activity toward the Gram-negative bacteria. Cadiolides C–F (**29**–**32**) was also showed antibacterial activities with methicillin-sensitive *S. aureus* (MSSA) and methicillin-resistant *S. aureus* (MRSA) ranging from 0.13 to 4 μg/mL of MICs [53]. In addition, it should be noted that none of these compounds showed significant cytotoxicity in the MTT assay at 100 μM. Therefore, cadiolides could serve as new lead compounds for the development of antibiotics for the treatment of bacterial infections caused by Gram-positive bacteria, such *as S. aureus* [53].

#### 3.1.21. Cytosporones B and E

**Figure 10:**
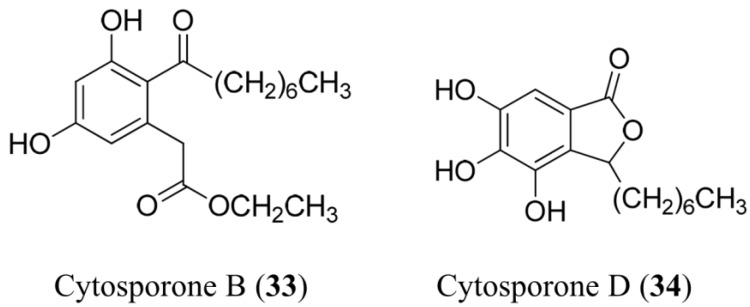
Structure of cytosporomes B (**33**) and E (**34**). Cytosporomes were isolated a strain of the endophytic fungus *Leucostoma persoonii* [54].

Cytosporomes B (**33**) and E (**34**) isolated a strain of the endophytic fungus *Leucostoma persoonii* from red mangrove, Rhizophora mangle [54] (Figure 10). Cytosporone B (**33**) demonstrated a 4.2-fold reduction in bacterial viability and at twice the MIC, resulted in complete killing of the bacteria. Furthermore, at MIC, a 2-fold reduction in biofilm formation was observed, and at twice the MIC, 168-fold reduction occurred. At higher concentrations, it appears strongly active toward biofilms, which is uncommon for antibiotics; however, cytosporone B is cytotoxic toward A549 cells (TI_90_ = IC_90_ A549/MIC_90_ = 6) [54]. Cytosporone E (**34**) was equipotent against USA100 and methicillin-sensitive *S. aureus* (MSSA) strains (72 μM), indicating the intrinsic drug resistant properties of MRSA strains are not helpful in resisting the action of this cytosporone. In addition, at MIC, it resulted in >5000-fold reduction in bacterial viability, indicating it is strongly bactericidal, and not just bacteriostatic. The cytosporone E MBC_90_ is significantly below its MIC, further demonstrating developmental potential. Cytosporone E is also cytotoxic, but reasonably selective for bacteria relative to mammalian cells (TI_90_ = 10). Cytosporone E (**34**), however, displayed an IC_90_ of 13 μM, which represents significant selectivity (TI_90_ = 33) for a moderately potent antimalarial drug [54].

#### 3.1.22. Anthracimycin

**Figure 11:**
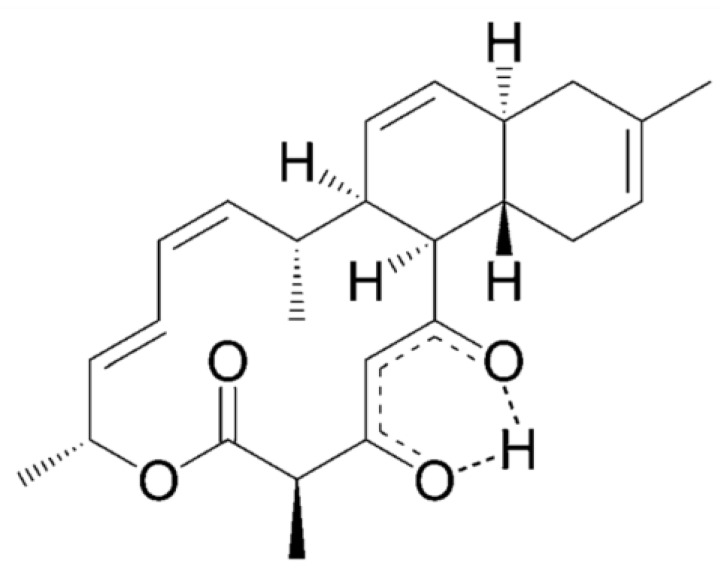
Structure of Anthracimycin (**35**). Anthracimycin were isolated from the *Steptomyces* bacteria [55].

Anthracimycin (**35**), a chemical compound derived from the *Steptomyces* bacteria, was discovered in the ocean off the coast of Santa Barbara in California [55]. It has shown significant activity against *B. anthracis*, *Enterococcus faecalis*, *Streptococcus pneumo* nia and *S. aureus* including MSSA, MRSA and vancomycin-resistant strains of *S. aureus* at MIC value between 0.03125 and 0.25 μg/mL. At concentrations near the MIC, anthracimycin inhibited *S. aureus* nucleic acid synthesis as determined by optimized macromolecular synthesis methodology, with inhibition of DNA and RNA synthesis occurring in the absence of DNA intercalation. Anthracimycin at a single dose of 1 or 10 mg/kg was able to protect mice from MRSA-induced mortality in a murine peritonitis model of infection. Anthracimycin provides an interesting new scaffold for future development of a novel MRSA antibiotic [55] (Figure 11).

### 3.2. Antifungal Activity

Pathogenic fungi cause diseases in humans and other organisms. *Candida* species are important human pathogens that are best known for causing opportunistic infections in immunocompromised hosts (e.g., transplant, AIDS, and cancer patients). *Aspergillus* can cause disease through the production of mycotoxins through the induction of allergenic responses and through localized or systemic infections. *Aspergillus flavus* produces aflatoxin, which is a carcinogen. *Cryptococcus neoformans* can cause severe forms of meningitis and meningo-encephalitis in patients with HIV infection and AIDS [97]. Fungal infections in humans range in severity from easily treated superficial infections of the skin and hair to life threatening disseminated infections that involve multiple organ systems. Among fungal infections (mycoses), crytococcosis and candidiasis, whether systemic (affecting deeper tissues and organs) or superficial (affecting skin, nails, scalp or mucous membrane), are serious problems in public health. Although fungi have existed for millions of years, their role in causing infections in humans was not recognized until the 1830s to 1840s. Early treatment modalities [98] of human infections were based on the plant mycologists’ experience, and included the use of toxic reagents, such as copper, mercury, iodide, Whitefield’s ointment (a combination of benzoic and salicylic acids), selenium sulfide, castellani paint, and gentian violet [97]. Griseofulvin, amphotericin B, allyl amines, and azole antifungals were developed during the 1950s to 1980s. Azole antifungals are remarkable as a drug class for their broad-spectrum targeting, oral bioavailability, and lower toxicity, as compared to other antifungals, such as polyenes (e.g., nystatin) and pyrimidines (e.g., 5-flucytosine). Nevertheless, new compounds being developed as resistance to azoles is slowly emerging, particularly in *C. albicans*. Diverse protein classes have emerged as novel, potent natural therapeutics. Prominent among metabolites with wide bioactivities in recent years are cyclic peptides, especially members of echinocandin family [99]. This suggests that, in principle, new antifungal agents based on peptidic structures are developing. From 2006 to the present, 18 studies reported antifungal marine peptides isolated from a diverse group of marine bacteria, ascidians, sponges and mollusk.

#### 3.2.1. Halocidin

**Figure 12:**
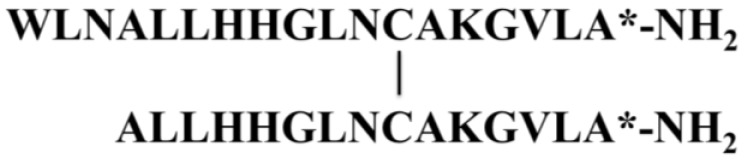
Amino acid sequence of halocidin (**36**). Halocidin were identified from the hemocytes of *Hyas araneus* [56]. Vertical bars in the sequence indicate a disulfide bond between two cysteine residues. The asterisk (*) denotes *C*-terminal amidation.

Jang* et al.* found that a synthetic analog of halocidin (**36**), a previously reported antimicrobial peptide isolated from the hemocytes of a marine ascidian, had potent antifungal activity (MIC = 1–4 μg/mL) [56]. The synthetic Di-K19Hc peptide was shown to bind *C. albicans* rapidly (30 s) via an interaction with β-1,3-glucan, a component of the fungal cell wall, and concomitantly inducing ion channel formation, K^+^ efflux, and death of the fungal cell. Halocidin is composed of two subunits, containing 18 and 15 amino acid residues joined by a single disulfide bond (Figure 12). An antimicrobial assay performed with synthetic congeners of halocidin containing the 18-residue monomer, showed that this subunit is more active than the heterodimer or the 15-residue monomer against MRSA and multi-drug resistant *P. aeruginosa* [100].

#### 3.2.2. Callipeltins J and K

Callipeltins are a group of marine peptides with unusual structural features and remarkable biological properties, isolated from the sponges *Callipelta* sp. [57]. From a structural point of view, the most distinctive feature of callipeltins is the presence of several non-proteinogenic units. Callipeltins K (**37**) and J (**38**) inhibit the growth of *C. albicans* ATCC 24433 in a standard disk assay with MIC value of 10^−4^ M. The antibiotic mixture selectively inhibited the growth of fungus, among which *C. albicans* (MIC = 1 μM) was the most affected [57].

#### 3.2.3. Pedein A

The cyclopeptide pedein A (**39**) is an antifungal peptides from the myxobacterium *Chondromyces pediculatus*. These cyclic peptides are notably composed of (6-chloro)-Trp, Gly, and several unusual amino acids. Pedein A inhibits many yeasts and fungi, especially *C. albicans* and *Rhizopus arrhizus*. (MIC = 0.6–1.6 μg/mL) [58]. Hopefully, future studies will evaluate the mechanism of action of these marine compounds.

#### 3.2.4. Theonellamide

Nishimura and colleagues evaluated the pharmacology of the bicyclic antifungal dodecapeptide theonellamide F (**40**), previously isolated from a sponge *Theonella* sp. [59]. Chemical-genomic profiling analysis, together with detailed subcellular localization studies, determined that the antifungal theonellamides represent a new class of sterol-binding molecules that induce membrane damage and activate *Rho1*-mediated 1,3-β-D-glucan synthesis.

Theonellamide G (**41**) showed potent antifungal activity towards wild and amphotericin B-resistant strains of *C. albicans*, with IC_50_s of 4.49 and 2.0 μM, respectively. Additionally, it displayed cytotoxic activity against the human colon adenocarcinoma cell line (HCT-16) with an IC_50_ of 6.0 μM. These findings provide further insight into the chemical diversity and biological activities of this class of compounds [60].

#### 3.2.5. Theopapuamides

Theopapuamides B (**42**) and C (**43**) were isolated from the Indonesian marine sponge *Siliquariaspongia mirabilis* [61]. Theopapuamide B (**42**) was active in a neutralization assay, with an IC_50_ value of 0.8 ± 0.3 μg/mL. Theopapuamides B and C showed cytotoxicity against human colon carcinoma (HCT-116) cells with IC_50_ values between 2.1 and 4.0 μg/mL, and exhibited strong antifungal activity against wild-type and amphotericin B-resistant strains of *C. albicans* at loads of 1–5 μg/disk [61].

#### 3.2.6. Surfactin

**Figure 13:**
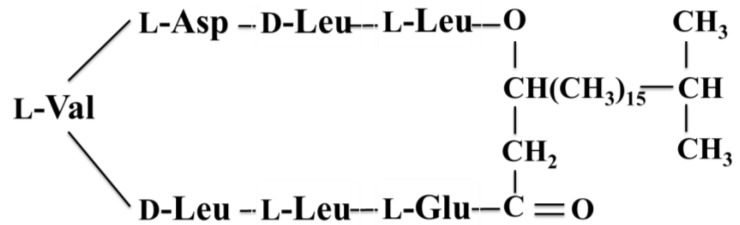
Structure of C(15)-surfactin (**44**). Surfactin was isolated from *Bacillus amyloliquefaciens* [101].

Surfactin is a lipopeptide biosurfactant with a broad spectrum of antimicrobial and antiviral activity. Surfactins have a peptide backbone composed of seven amino acids connected to a β-hydroxy fatty acid, which may vary from C-10 to C-16 [62] (Figure 13).

C(15)-surfactin (**44**) from *B. amyloliquefaciens* (6.25 μg/mL) synergized with the antifungal drug ketoconazole (0.004 μg/mL) to inhibit *C. albicans* SC5314. Optimum production of C(15)-surfactin (134.2 mg/L) with a 1.52-fold increase was achieved by employing response surface methodology in shaker flask cultivation in medium [101].

#### 3.2.7. Anti-CA Cyclic Lipopeptide

Anti-CA (**45**) from the bacterium *B. amyloliquefaciens*, isolated from a mangrove system, manifested fungicidal activity against a clinical strain of *C. albicans*. The main bioactive substance was a cyclic lipopeptide containing a heptapeptide, Asp-Leu-Leu-Val-Val-Glu-Leu, and a 3-OH fatty acid with 15 carbon atoms. The lipopeptide also killed other yeast strains including *C. tropicalis*, *Metschnikowia bicuspidata*, *Saccharomyces cerevisiae*, and *Yarrowia lipolytica* [63].

#### 3.2.8. Maribasins A and B

Two new cyclic lipopeptides, maribasins A (**46**) and B (**47**), were obtained from the fermentation broth of the marine microorganism *B. marinus* B-9987, which was isolated from *Suaeda salsa* from the Bohai coastline of China. The compounds displayed the structures cyclo (d-Pro-l-Gln-l-Asn-l-Ser-d-Asn1-d-Tyr-d-Asn2-d-β-aminoisopentadecanoic acid) and cyclo (d-Pro-l-Gln-l-Asn-l-Ser-d-Asn1-d-Tyr-d-Asn2-d-β-aminoanteisopentadecanoic acid), respectively. The lipopeptides demonstrated activity against a spectrum of phytopathogenic fungi [64].

#### 3.2.9. Mojavensin A

Three lipopeptides, designated as mojavensin A (**48**) were isolated from the fermentation broth of *Bacillus mojavensis* B0621A. Mojavensin A was characterized by a peptide backbone of l-Asn(1), d-Tyr(2), d-Asn(3), l-Gln(4), l-Pro(5), d-Asn(6), l-Asn(7), and an anteiso-type of saturated β-fatty acid side chain. These lipopeptides displayed dose-dependent antifungal activity against a broad spectra of phytopathogens, as well as being weakly antagonistic to *S. aureus*. Moreover, they all displayed cytotoxic activities against a human leukemia cell line (HL-60) with IC_50_ values of 100, 100, and 1.6 μM, respectively [65].

#### 3.2.10. Kahalalides

Another well-characterized, unusual group of peptides are the kahalalides, a family of depsipeptides with variable size and peptide series, ranging from C31 (tripeptide) to C77 (tridecapeptide) and carrying different fatty acid chains [66]. Hill* et al.* suggested that the Hawaiian sea slug *Elysia rufescens* acquires kahalalide-producing microbes from the surface of Bryopsis, and then retains these microbes as symbionts. Kahalalides, especially kahalalide F (**49**), are active against several fungi, such as *C. albicans* (IC_50_ = 3.02 μM), *C. neoformans* (IC_50_ = 1.53 μM), *Mycobacterium intracellulare*, and *A. fumigates* (IC_50_ = 3.21 μM) [102]. Their chemical synthesis has made it possible to characterize structure-activity relationships, resulting in the synthesis of derivatives with increased biological activity.

#### 3.2.11. Miuraenamides

Marine myxobacteria are rare, culture-resistant microorganisms, several strains of which have been identified by research groups in Asia. *Paraliomyxa miuraensis*, a slightly halophilic myxobacterium discovered in Japan, produces the cyclic hybrid polyketide-peptide antibiotics known as miuraenamide A (**50**) and B (**51**) [67]. Miuraenamide A and B are cyclic depsipeptides that possess one halogenated *N*-methyl-Tyr, one dehydro-Phe, and one Ala. Miuraenamides A and B are active against several fungi, such as *Phytophthora capsici* (IC_50_ = 0.4 μM), *A. niger* (IC_50_ = 50 μM), *Rhizopus oryzae* (IC_50_ = 6.3 μM), and *C. rugosa* (IC_50_ = 12.5 μM) [68]. The mechanism of miuraenamide synthesis has not yet been elucidated.

#### 3.2.12. Callyaerin

Callyaerins are cytotoxic cyclic peptides from the Indonesian marine sponge *Callyspongia aerizusa* [69]. Callyaerin A (**52**) exhibited strong inhibitory properties towards *C. albicans* and moderate activity against gram-negative *E. coli*, while it was only mildly active or inactive against gram-positive *S. aureus* and *B. subtilis*, respectively. Callyaerin E (**53**) displayed strong antimicrobial activity towards *C. albicans* and *B. subtilis*, but was only mildly active against the two remaining bacterial strains [69].

### 3.3. Anti-Malarial Activity

Malaria is the most threatening parasitic infection in humans. Each year, between 300 and 500 million new clinical cases are reported by the World Health Organization [103], resulting in annual deaths of approximately one million people [104], of which 75% are children below five years of age [105]. The most exposed continent is Africa; however, Southeast Asia, Oceania, and Central and South America are also under severe threat. Among the most affected are developing nations, which suffer from health care systems that lack the necessary infrastructure to cope with such diseases.

There are four species of *Plasmodium* infecting humans: *Plasmodium falciparum*, *Plasmodium vivax*, *Plasmodium ovale*, and *Plasmodium malariae*, but most malaria cases are caused by the protozoan parasite *P. falciparum* [106]. The parasite is transmitted by Anopheles mosquitos. Therefore, malaria can be controlled, to some extent, by conventional prevention strategies, such as mosquito repellents, mosquito traps, insecticides, or biological control. The introduction of efficient vaccines is still problematic, despite recent progress in this area [107]. As a direct consequence, the treatment of choice remains parasite chemotherapy with small molecule drugs, both of natural origin or synthetic [108,109]. In parallel, resistance to commonly used anti-malarial therapeutics is growing. Thus, there is an urgent need to develop new drugs against this disease. Moreover, combination treatment should be explored to reduce the risk of resistance. For example, combinations of artemisinin-amodiaquine and the artemether-lumefantine (Coartem) have been used. Contributing to the global search for novel antimalarial peptides, and as presented in Table 1, eleven novel marine peptides were shown to possess antimalarial activity between 2006 and the present.

#### 3.3.1. Dragomabin

McPhail* et al.* reported two marine cyanobacterial metabolites [70]. New linear alkynoic lipopetides, dragomabin (**54**) and dragonamide B, have been isolated from a red Panamanian strain of the marine cyanobacterium *Lyngbya majuscula* [65]. Dragomabin (**54**) showed good antimalarial activity (IC_50_ = 6 μM) against chloroquine-resistant *Plasmodium falciparum*, whereas the nonaromatic analogue, dragonamide B, was inactive.

#### 3.3.2. Venturamides

Linington* et al.* [71] discovered the new cyclic hexapeptides venturamide A (**55**) and B (**56**), which were isolated from a Panamanian marine cyanobacterium *Oscillatoria* sp. These modified cyclic hexapeptides showed moderate selectivity for parasites* versus* mammalian host cells. Venturamide A (**55**) showed* in vitro* activity against W2 chloroquinone-resistant *Plasmodium falciparum* (IC_50_ = 8.2 μM), with only mild cytotoxicity to mammalian Vero cells (IC_50_ = 86 μM). Venturamide B (**56**) also showed low micromolar antimalarial activity against *Plasmodium falciparum* (IC_50_ = 5.6 μM) and mild cytotoxicity to mammalian Vero cells (IC_50_ = 56 μM). The mode of action of these compounds is yet unknown.

#### 3.3.3. Aerucyclamides

Four new haxacyclopeptides, aerucyclamides A–D (**57**–**60**), were isolated from the cyanobacterium *Microcystis aeruginosa* PCC 7806, and their structural characterization, synthesis, and biological activity have been reported [72,73]. All four aerucyclamides were evaluated for anti-plasmodial activity. The most active compound was aerucyclamide B (**58**), which displayed a submicromolar IC_50_ value of 0.7 μM against the chloroquine-resistant *Plasmodium falciparum* strain K1. In addition, this compound displays a large selectivity for the parasite in L6 rat myoblasts, with an IC_50_ value of 120 μM. Interestingly, a reduction of the thiazole to a thiazoline (structural modification from A to B) decreases the antiplasmodial activity by 1 order of magnitude. Similar aerucyclamides C (**59**) and D (**60**) (IC_50_ = 2.3 and 6.3 μM, respectively) had low micromolar activities.

#### 3.3.4. Gallinamide A

Portmann* et al.* purified a novel linear peptide, gallinamide A (**61**), from a cyanobacterium *Schizothrix* sp. with an unusual 4-(*S*)-amino-2-(*E*)-pentenoic acid and methylmethoxy-pyrrolinone at the *C*-terminus [74]. Gallinamide A was moderately active against the chloroquinone-resistant *Plasmodium falciparum* strain W2 and *Leishmania donovani* (IC_50_ = 8.4 μM). Further, its structure has been deemed an “attractive foundation for further SAR investigations” [74].

#### 3.3.5. Lagunamide A and B

Tripathi* et al.* found two new cyclic depsipeptides, lagunamides A (**62**) and B (**63**), in the Singaporean marine cyanobacterium *Lyngbya majuscule*, which had potent antimalarial activity (IC_50_ = 0.19–0.91 μM) against the drug-sensitive *Plasmodium falciparum* strain NF54 [75].

#### 3.3.6. Albopunctatone

**Figure 14:**
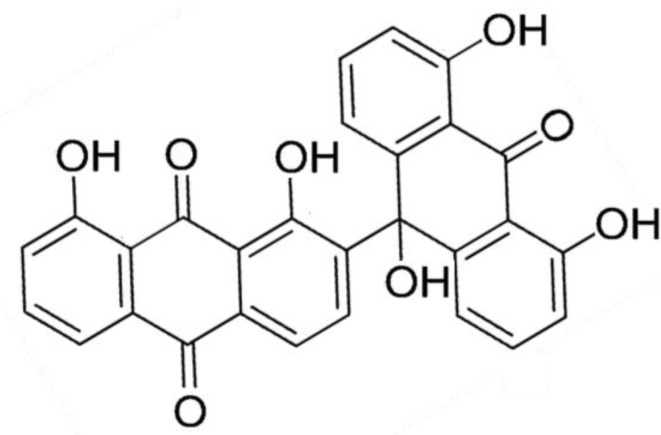
Structure of albopunctatone (**63**). Albopunctatone was isolated ascidian *Didemnum albopunctatum* [76].

Carroll* et al.* isolated a new anthrone-anthraquinone, albopunctatone (**64**) in the MeOH extract of the Great Barrier Reef ascidian *Didemnum albopunctatum*, which had moderately antimalarial activity (IC_50_ = 5.3 and 4.4 ± 0.5 μM) against chloroquine-resistant (Dd2) and -sensitive strains (3d7) of *Plasmodium falciparum* [76]. Albopunctatone (**64**) was also inactive up to 40 μM when tested against a variety of cancerous and normal human cell lines and the kinetoplastid *Trypanosoma brucei brucei*, indicating selectivity for the malaria parasite, *P. falciparum* [76] (Figure 14).

### 3.4. Antiprotozoal Activity

Seventeen marine peptides were reported to possess antimicrobial activity against other protozoa, thus contributing to the ongoing global search for novel agents to treated so-called neglected diseases, including leishmaniasis (caused by several species of the genus *Leishmania*), amoebiasis, trichomoniasis, African sleeping sickness (caused by *Trypanosoma brucei rhodesiense* and *Trypanosoma brucei gambiense*) and American sleeping sickness or Chagas disease (caused by *Trypanosoma cruzi*).

#### 3.4.1. Viridamides

An investigation of the marine cyanobacterium *Oscillatoria nigro-viridis* from Panama led to the isolation of two novel bioactive lipopeptides, viridamides A (**65**) and B (**66**) [77]. Viridamide A was tested against a series of relevant tropical pathogens and cancer cell lines, and displayed significant activity against the parasitic protozoan *Plasmodium falciparum* (IC_50_ = 5.8 μM). Further, viridamide A also exhibited activity against *Leishmania mexicana* (IC_50_ = 1.1 μM) and *Trypanosoma cruzi* (IC_50_ = 1.5 μM).

#### 3.4.2. Almiramides

Sanchez* et al.* isolated two novel linear lipopeptides, almiramides B (**67**) and C (**68**), from the marine cyanobacterium *Lyngbya majuscule*. Almiramides B and C displayed strong antiparasitic activity against *Leishmania donovani* (IC_50_ = 1.9–2.4 μM) with minimal cytotoxicity towards Vero cells (IC_50_ = 33.1–52.3 μM) [78], whereas Almiramides A was inactive up to 13.5 μM, indicating the requirement of an unsaturated terminus in the side chain for the activity [78].

#### 3.4.3. Valinomycin

Pimentel-Elardo* et al.* reported the isolation of the known cyclic depsipeptide valinomycin (**69**) from marine *Streptomyces* sp. strains associated with several Croatian marine sponges, and observed significant activity against both *Trypanosoma brucei* (IC_50_ = 0.0032 μM) and *Leishmania major* (IC_50_ < 0.11 μM) [79].

#### 3.4.4. Diketopiperazines

**Figure 15:**
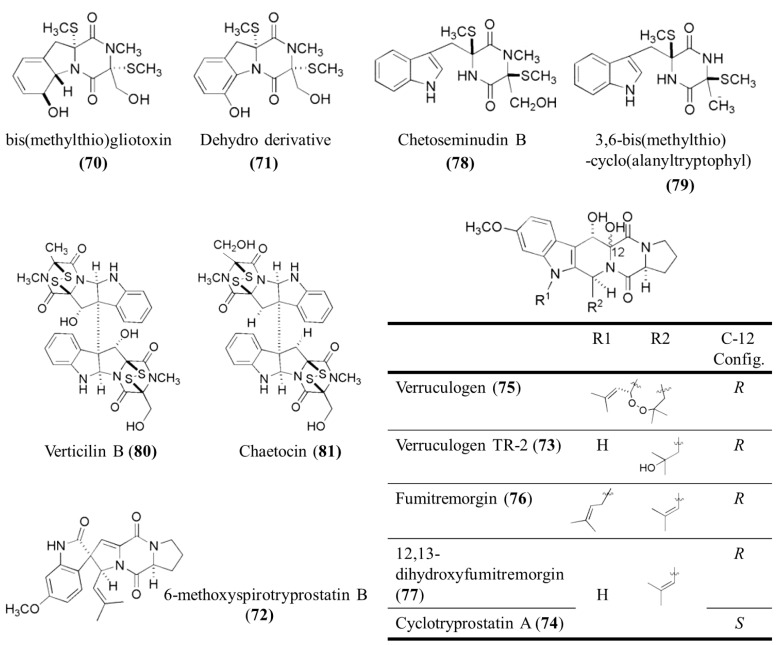
Structure of diketopiperazines (**70**–**81**) [80].

Watts* et al.* reported the crude extracts of two fungal strains (*A. fumigatus* and *Nectria inventa*) isolated from deep water sediment which provided >99% growth inhibition at 1 μg/mL of *Trypanosoma brucei*, the causative parasite of Human African trypanosomiasis (HAT, commonly known as African sleeping sickness) [80]. The saltwater *Aspergillus* culture presented five compounds: bis(methylthio)gliotoxin (**70**), its dehydro derivative (**71**), 6-methoxyspirotryprostatin B (**72**), verruculogen TR-2 (**73**), and cyclotryprostatin A (**74**). Unexpectedly, the deionized water culture of this strain produced three different metabolites: verruculogen (**75**), fumitremorgin B (**76**) and 12,13-dihydroxyfumitremorgin C (**77**). The deionized water culture of *N. inventa* was a source of four sulfur-containing nitrogen-containing diketopiperazines: chetoseminudin B (**78**), 3,6-bis(methylthio)-cyclo(alanyltryptophyl) (**79**), verticillin B (**80**) and chaetocin (**81**) (Figure 15). Twelve of the compounds, each containing a diketopiperazine core, showed excellent activity against *T. brucei* (IC_50_ = 0.002–40 μM), with selectivity over mammalian cells as great as 20-fold [80]. The trypanocidal diketopiperazines were also tested against two cysteine protease targets. Rhodesain and TbCatB, where five compounds showed inhibition activity at concentrations less than 20 μM. [80].

### 3.5. Antituberculosis Activity

Antituberculosis agents are drugs used to treat tuberculosis, an infectious disease caused by *Mycobacterium tuberculosis*. This infection mainly affects the lungs, but can also affect many other organ systems. Many classes of drugs with different mechanism of action have activity against *M. tuberculosis*. As shown in Table 1, one paper (three compounds) reported work regarding anti-tuberculosis pharmacology with marine peptides from 2006 to the present.

#### Trichoderins

Three new aminolipopeptides, trichoderins A (**82**), A1 (**83**), and B (**84**), were isolated from a culture of the marine sponge-derived fungus *Trichoderma* sp. as anti-mycobacterial substances with activity against active and dormant bacilli [106]. Trichoderins showed potent antimycobacterial activity against *Mycobacterium smegmatis*, *Mycobacterium bovis*, and *Mycobacterium tuberculosis* under standard aerobic growth conditions, as well as dormancy-inducing hypoxic conditions, with MIC values in the range of 0.02–2 μg/mL [81].

### 3.6. Anti-Viral Activity

Influenza continues to be a danger to human and animal health. The ability of the virus to undergo rapid mutation means that antigenic variants constantly arise, breeching the host-adaptive immune response. Pathogenic variants may arise at any time, and through reassortment, can generate new subtypes with the potential to cause pandemics. Approaches to broaden the availability of novel antiviral compounds include the development of synthetic peptides that disrupt the entry of the virus into cells. Antiviral peptides have been developed against hepatitis C [110], West Nile virus [111], respiratory syncytial virus [112], human immunodeficiency virus (HIV) [113], and the influenza virus [114]. The clinical application of the peptide Enfuvirtide (FUZEON), which blocks the entry of HIV into cells, points to the success of peptide-based therapies in a clinical setting. Other key therapeutic peptides include bivalirudin (Angiomax), a platelet aggregation inhibitor peptide, and abarelix (Plenaxis), a gonadotropin-releasing hormone (GnRH) antagonist. These are typical of the new generation of peptides with excellent therapeutic efficacy.

As shown in Table 1, thirty reports were published from 2006 to the present on the antiviral pharmacology of novel marine peptides against human cytomegalovirus and herpes simplex virus. Three articles reported preclinical pharmacology of marine compounds active against the human immunodeficiency virus type-1 (HIV-1), the causative agent of the acquired immunodeficiency disease syndrome (AIDS).

#### 3.6.1. Mirabamides A, C, and D

Plaza* et al.* described three cyclic depsipeptides, mirabamides A (**85**), C (**86**) and D (**87**), isolated from the sponge *Siliquariaspongia mirabilis* [82]. Mirabamides contain two entities, including 4-chloromoproline in 1–3 and an unusual glycosylated amino acid, β-methoxytyrosine 4′-*O*-α-L-rhamnopyranoside (in 1, 2 and 4), along with a rare *N*-terminal aliphatic hydroxy acid. Mirabamide A (**85**) inhibited HIV in neutralization and fusion assays with IC_50_ values between 40 and 140 nM, as did mirabamides C (**86**) and D (**87**) (IC_50_ values between 140 nM and 1.3 μM for C and 190 nM and 3.9 μM for D), indicating that these peptides can act at the early stages of HIV-entry. Additionally, mirabamides A and C inhibited the growth of *B. subtilis* and *C. albicans* at 1–5 μg/disk in a disk diffusion assays [82].

Four other homogenous depsipeptides, mirabamides E–H (**88**–**91**), from the sponge *Stelletta clavosa* also showed strong inhibition of HIV-1 in a neutralization assay using the viral strain YU2-V3 with IC_50_ values of 121, 62, 68, and 41 nM, respectively [115]. The primary feature distinguishing mirabamides E–H from mirabamides A, C, and D was the presence of 2-amino-2-butenoic acid in place of threonine.

#### 3.6.2. Mollamide B

Mollamide B (**92**) is a cyclic hexapeptide isolated from the Indonesian tunicate *Didemnum molle*. Mollamide B exhibited moderate antimalarial activity against *P. falciparum* (D6 clone and W2 clone), with IC_50_ values of 2.0 and 2.1 μg/mL, respectively. Further, mollamide B also exhibited marginal activity against *Leishmania donovani*, with IC_50_ and IC_90_ values of 18 and 35 μg/mL, respectively, and against HIV-1 in human PBM cells with an EC_50_ value of 48.7 μM [83].

Mollamide B was examined for antimicrobial activity against MRSA, *Mycobacterium intracellulaire*, *C. albicans*, *C. glabrata*, *C. krusei*, and *C. neoformans*, and exhibited no activity. Mollamide B has shown no anti-inflammatory activity in rat neonatal microglia, and did not inhibit cyclooxygenase-2 (COX-2) activity in a cell-based assay [116].

#### 3.6.3. Papuamides

Papuamides are a class of marine sponge-derived cyclic depsipeptides, which are thought to have cytoprotective activity against HIV* in vitro* through the inhibition of viral entry [84]. Papuamide A (**93**) is a novel marine compound isolated from the sponge *Theonella* sp. Papuamide A was tested in a fusion assay and against the HCT-116 cell line, with IC_50_ values of 73 nM and 3.5 μM. The anti-HIV activity of papuamide was shown to occur through a membrane-targeting mechanism, in which the hydrophobic tail of the molecule inserts into the viral membrane and the tyrosine residue interacts with cholesterol [84].

#### 3.6.4. Celebesides A and C

Plaza* et al.* isolated several new cyclic depsipeptides from the Indonesian marine sponge *Siliquariaspongia mirabilis*, including celebesides A (**94**) and C (**95**), which inhibited HIV-1 in an infectivity assay (IC_50_ = 1.9 ± 0.4 μg/mL) [61]. Celebeside A was active against both HIV-1 (IC_50_ = 0.002 μM) and HCT 116 cancer cells (IC_50_ = 0.009 μM) [61]. The lipid component of celebeside A is of mixed origin. Celebesides A and C were tested against HCT-116 cells, giving IC_50_ values of 9.9 and >31 μM, respectively. The ability to inhibit HIV-1 entry was also evaluated for both celebeside A and C, with IC_50_ values of 2.1, and >62 μM, respectively. Interestingly, for both celebesides A and C in biological assays, loss of activity was correlated with the loss of the phosphate group.

#### 3.6.5. Theopapuamides

Theopapuamide A (**96**) is a cytotoxic undecapeptide isolated from *Theonella swinhoei* collected in Milne Bay, Papua New Guinea [85]. It is the first natural peptide containing β-methoxyasparagine and 4-amino-5-methyl-2,3,5-trihydroxyhexanoic acid residues. It was tested in the CEM-TART (T-cells that express both HIV-1 tat and rev) and HCT-116 colorectal carcinoma cell lines with IC_50_ values of 0.5 and 0.9 μM, respectively.

Plaza* et al.* isolated cyclic peptides, theopapuamides B (**42**), C (**43**), and D (**97**), from an extract of *Siliquariaspongia mirabilis* collected off Sulawesi Island, Indonesia [61]. Theopapuamides B (**41**) and C (**42**) were tested against HCT-116 cells giving IC_50_ values of 2.5 and 1.3 μM, respectively. The ability to inhibit HIV-1 entry was also evaluated for theopapuamides B (**42**), with IC_50_ values of 0.5 μM. Theopapuamides A (**96**), B (**42**), and C (**43**) were evaluated for their ability to inhibit the growth of both wild type and amphotericin B-resistant strains of *C. albicans*. Theopapuamide A inhibited the growth of both strains with zones of inhibition of 8 mm at 1 μg/disk, while theopapuamides B and C displayed zones of 10 mm against both strains at 5 μg/disk [61].

#### 3.6.6. Asperterrestide A

Asperterrestide A (**98**), a cyclic tetrapeptide with a rare 3-OH-*N*-CH_3_-Phe residue, was isolated from the fermentation broth of the marine-derived fungus *Aspergillus terreus* SCSG AF0162. Asperterrestide A showed inhibitory effects on influenza virus strains A/WSN/33 (H1N1) (an M2-resistant strain) and strain A/Hong Kong/8/68(H3N2) (an M2-sensitive strain), with IC_50_ values of 20.2 and 0.41 μM, respectively. Cytotoxic activity was tested in U937 and MOLT4 human carcinoma cell lines with IC_50_ values of 1.9 and 1.8 nM, respectively [86].

#### 3.6.7. Homophymine A

Homophymines A–E (**99**–**103**) and A1–E1 (**104**–**108**) are a series of cyclodepsipeptides isolated from *Homophymia* sp. collected from shallow waters off the east coast of New Caledonia [87,88]. The anti-viral properties of Homophymines A were tested in an assay with peripheral blood mononuclear cells (PBMC) infected with the III B strain of HIV-1. Homophymine A had cytoprotective properties through the inhibition of infection production, with an IC_50_ value of 75 nM. Homophymine A was cytotoxic against uninfected PBMC cells with an IC_50_ of 1.19 μM, but it was almost sixteen times more effective against infected cells. Homophymines A–E and A1–E1 were evaluated against a panel of cell lines including human cancer and the Vero green monkey kidney cell lines. Homophymines A–E and A1–E1 exhibited potent cytotoxicity with IC_50_ values ranging from 2 to 100 nM. They were the most potent in the PC3 human prostate adenocarcinoma and the SK-OV3 human ovarian adenocarcinoma cell lines [87].

#### 3.6.8. Koshikamide

**Figure 16:**
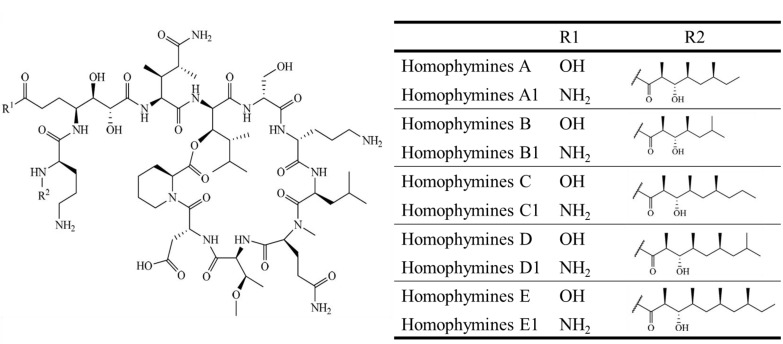
Structure of Homophymines A–E (**99**–**103**) and A1–E1 (**104**–**108**). Homophymines are a series of cyclodepsipeptides isolated from *Homophymia* sp. collected from shallow waters off the east coast of New Caledonia [87,88].

The cytotoxic cyclic peptide lactone, koshikamide B (**109**), is the first account of a peptide possessing a carbamoylated asparagine and the new amino acid residue 2-(3-amino-2-hydroxy-5-oxopyrrolidin-2-yl) propionic acid (AHPP) [89]. It was initially isolated from a *Theonella* sp., collected off Shimokoshiki Island, Kagoshima Prefecture, Japan. It exhibited cytotoxicity against P388 murine leukemia and HCT-116 human colon tumor cell lines, with IC_50_ values of 0.22 and 3.7 μM, respectively (Figure 16). Koshikamides F–H (**110**–**112**) are 17-residue depsipeptides containing a 10-residue macrolactone that inhibits HIV-1 entry against a CCR5-using viral envelope, with IC_50_ values of 2.3 and 5.5 μM, respectively, whereas their linear kishikamides C were inactive [90]. Koshikamides F–H also had moderate cytotoxicity in the HCT-116 colon cancer cell line with an IC_50_ of 10 μM. Koshikamides F-H did not inhibit the growth of *C. albicans*.

## 4. Conclusions

Although marine peptides are largely unexplored, they have inherent activity and an ability to inhibit infection. Marine peptides are structurally diverse, have a wide spectrum of therapeutic action, a low bio-deposition rate in body tissues, and are highly specific to targets. Marine derived peptides also possess reduced risk of unwanted adverse side-effects. As marine peptides are composed of metabolically tolerable amino acids, they are generally safe and non-toxic. In addition to their use as active ingredients, marine peptides also have the ability to be used as excipients in drug formulations for the modification of biological activity, targeted delivery, or transport across cellular membranes. There are also a number of issues that have to be considered from the earliest stages of development when a marine chemical entity is selected for clinical development, including, the effects, sourcing, technological and scientific difficulties, and legal uncertainties. Finding new anti-infective peptides in marine living resources, particularly those living in deep-sea and special marine environments, is an important approach to identify novel active agents. Moreover, screening anti-infective activities in the existing marine peptide library, as well as semi-synthetic modifications of anti-infective agents, can be used to identify novel compounds. Extensive studies of anti-infective peptides will contribute to the generation of novel pharmaceutical products. Thus, marine peptides are a valuable source of bioactive compounds, which could be introduced for development in pharmaceutical industries.
